# Sulfuric Odor Precursor *S*-Allyl-l-Cysteine Sulfoxide in Garlic Induces Detoxifying Enzymes and Prevents Hepatic Injury

**DOI:** 10.3390/antiox8090385

**Published:** 2019-09-10

**Authors:** Yusuke Yamaguchi, Ryosuke Honma, Tomoaki Yazaki, Takeshi Shibuya, Tomoya Sakaguchi, Harumi Uto-Kondo, Hitomi Kumagai

**Affiliations:** 1 Department of Chemistry and Life Science, Nihon University, 1866 Kameino, Fujisawa-shi 252-0880, Japan; 2 Department of Bioscience in Daily Life, Nihon University, 1866 Kameino, Fujisawa-shi 252-0880, Japan

**Keywords:** organosulfur compound, odor precursor, garlic, hepatic injury, Nrf2

## Abstract

*S*-Allyl-l-cysteine sulfoxide (ACSO) is a precursor of garlic-odor compounds like diallyl disulfide (DADS) and diallyl trisulfide (DATS) known as bioactive components. ACSO has suitable properties as a food material because it is water-soluble, odorless, tasteless and rich in bulbs of fresh garlic. The present study was conducted to examine the preventive effect of ACSO on hepatic injury induced by CCl_4_ in rats. ACSO, its analogs and garlic-odor compounds were each orally administered via gavage for five consecutive days before inducing hepatic injury. Then, biomarkers for hepatic injury and antioxidative state were measured. Furthermore, we evaluated the absorption and metabolism of ACSO in the small intestine of rats and NF-E2-related factor 2 (Nrf2) nuclear translocation by ACSO using HepG2 cells. As a result, ACSO, DADS and DATS significantly suppressed the increases in biomarkers for hepatic injury such as the activities of aspartate transaminase (AST), alanine transaminase (ALT) and lactate dehydrogenase (LDH), and decreases in antioxidative potency such as glutathione (GSH) level and the activities of glutathione *S*-transferase (GST) and glutathione peroxidase (GPx). We also found ACSO was absorbed into the portal vein from the small intestine but partially metabolized to DADS probably in the small intestine. In in vitro study, ACSO induced Nrf2 nuclear translocation in HepG2 cells, which is recognized as an initial trigger to induce antioxidative and detoxifying enzymes. Taken together, orally administered ACSO probably reached the liver and induced antioxidative and detoxifying enzymes by Nrf2 nuclear translocation, resulting in prevention of hepatic injury. DADS produced by the metabolism of ACSO in the small intestine might also have contributed to the prevention of hepatic injury. These results suggest potential use of ACSO in functional foods that prevent hepatic injury and other diseases caused by reactive oxygen species (ROS).

## 1. Introduction

Sulfur is one of the key elements involved in the regulation of biological functions in the human body. Pivotal roles of organosulfur compounds are to maintain redox balance and to detoxify toxic agents. Reduced glutathione (GSH) is ubiquitously expressed in cells and reduces oxidative agents, such as hydroxyl radicals, oxide anion radicals, and hydrogen peroxide [1,2], playing a central role in detoxification [3,4]. Although GSH is important for such defense, oral intake of GSH does not necessarily increase these antioxidative and detoxifying activities in the human body [5,6]. Orally administered GSH can be delivered to various organs of the human body [7]; however, the effects of GSH are cancelled by the metabolite l-cysteine-l-glycine (Cys-Gly), which serves as a pro-oxidant to readily produce thiyl radicals and reactive oxygen species (ROS), resulting in oxidative stress [8]. Therefore, in order to reduce oxidative stress via oral administration of a natural compound, it is not sufficient for a functional compound to merely reach the target organ. The compound needs to increase the antioxidative and detoxifying activities by regulating transcription of biomolecules involved in these activities as electrophiles in food [9,10]. In addition, for maximum effectiveness, the metabolites of the compound are also required to increase the antioxidative and detoxifying activities.

**Figure 1:**
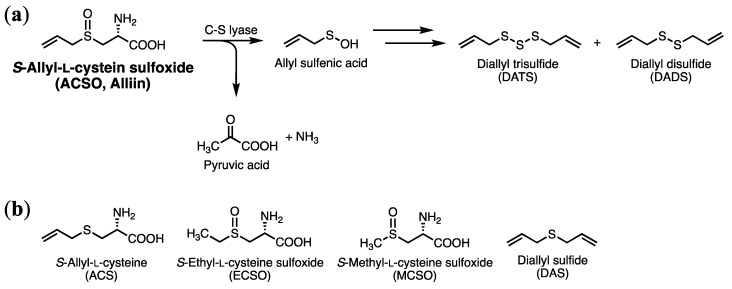
Production of garlic odor molecules from *S*-allyl-l-cysteine sulfoxide (ACSO) (**a**) and its related compounds tested in this study (**b**).

Garlic has been used as a medicinal food since ancient times. This plant is reported to exert anti-cancer [11,12,13], anti-atherosclerotic [14], anti-diabetic [15], anti-tumoral [16], anti-bacterial [17], antioxidative [18], and detoxifying [19] activities in animal studies. Most of these effects have been attributed to organosulfur compounds, such as diallyl disulfide (DADS) and diallyl trisulfide (DATS), which have distinctive garlic odors produced when the garlic bulb is crushed or sliced [20]. During these processes, a garlic odor precursor, *S*-allyl-l-cysteine sulfoxide (ACSO; also known as alliin) in the cytoplasm collides with cysteine *S*-conjugate beta-lyase (C-S lyase) that leaked from the vacuole, producing garlic odor compounds along with pyruvic acid (Figure 1). Although studies have demonstrated that the physiological effects of garlic are due to the odor components DADS and DATS, odor precursors may also contribute to these effects. Indeed, some previous studies have reported that ACSO exerts anti-diabetic [21], anti-myocardial ischemia [22], hypoglycemic [23], and hypolipidemic effects [23] in animal studies. We have also reported that ACSO inhibits platelet aggregation [24] and suppresses increases in blood ethanol concentration [25] in animal studies. Considering that raw garlic contains up to 14 mg ACSO/g fresh weight [26] and ACSO can be retained just by heating bulbs before cutting to inactivate enzymes, garlic extract or powder rich in ACSO can be easily prepared from preheated bulbs. In addition, as ACSO is odorless and works as a taste enhancer [27], ACSO or garlic with high ACSO level can be added to various foods. Therefore, if oral administration of ACSO and/or its metabolites effectively increases the antioxidative and detoxifying activities of garlic, ACSO or such ACSO-rich garlic material would be a promising functional-food additive to prevent diseases caused by oxidative stress.

Antioxidative and detoxifying effects of food components can be evaluated by examining its preventive effect on acute hepatic injury induced by carbon tetrachloride (CCl_4_) [28,29,30,31]. Intraperitoneally administered CCl_4_ is transported to the liver and reduced by phase I enzymes, such as CYP450, to yield trichloromethyl radical (CCl_3_ radical) [32,33], which forms covalent bonds with proteins, lipids, and nucleic acids to impair their functions [34,35]. In addition, CCl_3_ radical oxidizes lipids to produce lipid peroxide, which destroys the lipid bilayer of cell membranes, resulting in leakage of the liver cell contents, such as aspartate transaminase (AST), alanine transaminase (ALT), and lactate dehydrogenase (LDH), into the blood [36]. Therefore, CCl_4_-induced hepatic injury causes increases in the amounts of lipid peroxide in the liver and AST, ALT, and LDH activities in the blood. In order to prevent the symptoms of CCl_4_-induced hepatic injury, detoxification of the peroxide in vivo by phase II enzymes, including glutathione-*S*-transferase (GST) and quinone reductase (QR) and antioxidative enzymes such as glutathione reductase (GR) and glutathione peroxidase (GPx), should occur immediately [28,29,30,31]. GSH is consumed by detoxification with GST as well as the reduction of peroxide with GPx and the oxidation of GSH, while GSH is recovered by the reduction of oxidized GSH with GR [28,29,30,31]. Therefore, if intake of ACSO can prevent CCl_4_-induced hepatic injury, increases in AST, ALT, LDH, and lipid peroxide as well as decreases in the amount of GSH will be suppressed by the concomitant increases in GST, QR, GR and GPx activities. Phase II enzymes and antioxidative enzymes are induced by the activation of the NF-E2-related factor 2 antioxidant response element (Nrf2-ARE) pathway [37,38]. This activation is triggered by Nrf2 nuclear translocation. To activate the pathway in the liver, orally administered ACSO and/or its bioactive metabolites are required to be transported to the liver, inducing translocation of Nrf2 to the nucleus. 

The present study was conducted to examine the effect of oral administration of ACSO on the prevention of hepatic injury induced by CCl_4_. Furthermore, we investigated the mechanism underlying the preventive effect on CCl_4_-induced hepatic injury. We also evaluated Nrf2 nuclear translocation in liver cells following ACSO administration and the absorption of ACSO from the small intestine into the portal vein, which leads to the liver. Moreover, in order to examine whether intact ACSO is absorbed from the small intestine or metabolized during absorption, we also measured the ACSO and the ACSO metabolite pyruvic acid in the blood after injection of ACSO in the ligated loop of the small intestine in rats.

## 2. Materials and Methods 

### 2.1. Materials

*S*-Allyl-l-cysteine sulfoxide (ACSO), *S*-methyl-l-cysteine sulfoxide (MCSO), and *S*-ethyl-l-cysteine sulfoxide (ECSO) were synthesized from the corresponding alk(en)yl bromide and l-cysteine followed by the addition of hydrogen peroxide [25,39]. *S*-Allyl-l-cysteine (ACS), diallyl sulfide (DAS), diallyl disulfide (DADS), and diallyl trisulfide (DATS) were purchased from Tokyo Chemical Industry Co., Ltd. (Tokyo, Japan). Chemical reagents for the experiments were purchased from Wako Pure Chemical Industries, Ltd. (Osaka, Japan), Oriental Yeast Co., Ltd. (Tokyo, Japan), Cosmobio Co., Ltd. (Tokyo, Japan), and Roche Diagnostics GmbH Co., Ltd. (Mannheim, Germany).

### 2.2. In Vivo and Ex Vivo Experiments

#### 2.2.1. Animals

Seven-week-old male Sprague Dawley (SD) rats were purchased from Japan SLC, Inc. (Tokyo, Japan). All animal experiments were performed in accordance with the Guidelines for Animal Experiments of the College of Bioresource Sciences, Nihon University (approval code: AP13B010 and AP16B139). The feeding facility was maintained at an ambient temperature of 21–22 °C with 12-h light-dark cycling. Rats were housed in individual stainless-steel wire cages with free access to food (CE-2, Clea Japan, Tokyo, Japan) and water during a 1-week-acclimation period prior to the experiments.

#### 2.2.2. Effect of ACSO and Its Related Compounds on Suppression of Hepatic Injury Induced by CCl_4_

**Figure 2:**
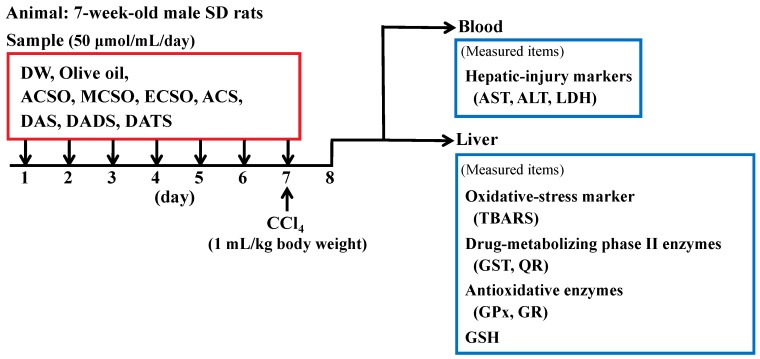
Animal experimental design for evaluation of the suppressive effects of *S*-allyl-l-cysteine sulfoxide (ACSO) and its related compounds on hepatic injury induced by CCl_4_.

Rats were divided into 11 groups of six rats (Figure 2). ACSO, MCSO, ECSO, ACS, DAS, DADS, and DATS at a dosage of 50 μmol/mL/day were orally administered via gavage to rats of the corresponding group for seven consecutive days. ACSO, MCSO, ECSO, and ACS were dissolved in distilled water (DW), while DAS, DADS, and DATS were dissolved in olive oil at the time of use. Control rats received only either DW or olive oil. CCl_4_ was intraperitoneally administered at a dosage of 1 mL/kg body weight after oral administration of the sulfoxides and sulfides on the seventh day. Then, the rats were subjected to fasting for 24 h and thereafter sacrificed. The liver was excised, and the microsomal and cytosolic fractions were prepared by centrifugation as described [40]. Briefly, serum was separated from sodium citrate-treated whole blood by centrifugation at 1500× *g* for 15 min at 4 °C. 

##### (a) Determination of AST, ALT and LDH Levels

The obtained serum in 2.2.2. was used to examine acute hepatic-injury enzyme markers (i.e., AST, ALT, and LDH). These levels in the serum were determined by the enzymatic method using an automatic analyzer, Spotchem EZ SP-4430 (Liver-2, Arkray, Inc., Kyoto, Japan).

##### (b) Measurement of Lipid Peroxide

The content of lipid peroxide in the liver was determined by thiobarbituric acid (TBA) reactive substances (TBARS) assay, which detects aldehydes produced from the decomposition of lipid hydroperoxide [41]. First, 0.5 mL of the liver homogenate was mixed with 0.3 mL of 1% phosphoric acid and 1 mL of 0.67% TBA aqueous solution. The mixture was incubated at 95 °C for 45 min to produce aldehyde-TBA adduct possessing absorbance at 535 nm. After cooling the reaction mixture to room temperature, 4 mL of *n*-butanol was added to dissolve the adduct. The mixture was centrifuged at 1500× *g* for 10 min, and the supernatant containing aldehyde-TBA adduct was obtained. The absorbance of the supernatant at 535 nm was measured. The difference in absorbance at 535 nm between the mixture with and without the adduct was used as the TBARS value. Malondialdehyde (MDA) was used as a standard and the TBARS value was expressed as MDA equivalent.

##### (c) Measurement of GST Activity

The activity of glutathione *S*-transferase (GST) in the cytosol of the liver was spectrophotometrically assayed [42]. A 1.48 mL solution containing 0.1 M potassium phosphate buffer at pH 7.4, 30 mM reduced glutathione (GSH) was preincubated at 25 °C for 5 min. Then, 60 μL of 30 mM 1-chloro-2,4-dinitrobenzene (CDNB) and 300 μL cytosol fraction were added to the solution to initiate the reaction of CDNB to produce *S*-2,4-dinitrophenylglutathione, and the increase in absorbance at 340 nm was recorded. The amount of *S*-2,4-dinitrophenylglutathione was calculated by its extinction coefficient (ε = 9.6 mM^−1^ cm^−1^). The activity of GST was expressed as the amount of *S*-2,4-dinitrophenylglutathione produced per minute per milligram of cytosol protein. The amount of cytosol protein was determined by the bicinchoninic acid (BCA) method using BCA Protein Assay Kit (Thermo Fisher Scientific, Waltham, MA, USA).

##### (d) Measurement of QR Activity

The activity of quinone reductase (QR) in the cytosol of liver was assayed spectrophotometrically [43]. An 800 μL solution containing 50 mM Tris-HCl at pH 8.0, 0.2% Tween-20, 40 μM oxidized 2,6-dichlorophenolindophenol (DCIP), and 0.3 mM NADPH was preincubated at 25 °C for 5 min. Then, 150 μL of cytosol fraction was added to the solution to produce reduced DCIP from oxidized DCIP. A decrease in the absorbance at 600 nm was recorded, and the activity of QR was calculated from the difference between the absorbance with and without the cytosol fraction. The amount of oxidized DCIP was calculated by its extinction coefficient (ε = 21 mM^−1^ cm^−1^). The activity of QR was expressed as the amount of oxidized DCIP consumed per minute per milligram of cytosol protein. The amount of cytosol protein was determined by the BCA method.

##### (e) Measurement of GR Activity

The activity of glutathione reductase (GR) in the liver was measured as described by Carlberg and Mannervik [44]. A 600 μL of solution containing 0.1 M potassium phosphate buffer at pH 7.6 containing 1 mM ethylenediaminetetraacetic acid (EDTA), 0.1 mM reduced nicotinamide-adenine dinucleotide phosphate (NADPH), 1 mM oxidized glutathione (GSSG), and 0.1% bovine serum albumin (BSA) was mixed with 100 μL of the cytosol fraction to produce GSH from GSSG. The decrease in absorbance of NADPH at 340 nm was monitored at 25 °C, and the amount of NADPH consumed was calculated by using its molar extinction coefficient (ε = 6.22 mM^−1^ cm^−1^). The activity of GR was expressed as the amount of NADPH consumed per minute per milligram of cytosol protein. The amount of cytosol protein was determined by the BCA method.

##### (f) Measurement of GPx Activity

The activity of glutathione peroxidase (GPx) in the liver was determined spectrophotometrically using GSH and hydrogen peroxide (H_2_O_2_) as substrates [45]. First, 20 μL of 0.1 M GSH, 100 μL of 10 unit/mL GR, and 100 μL of 2 mM NADPH were mixed with 100 μL of 0.1 M sodium phosphate buffer and 2 mM NaN_3_ at pH 7.0 in a sample cuvette. Then, 10 μL of the cytosol fraction was added to the mixture, while 10 μL of buffer was added to the reference cuvette. The total volume of the solution was adjusted to 1 mL by adding 660 μL of DW into each cuvette. After preincubation at 37 °C for 2 min, the reaction was started by adding 10 μL of 1.5 mM H_2_O_2_. The oxidation of NADPH to oxidized nicotinamide-adenine dinucleotide phosphate (NADP^+^) along with the conversion of GSSG to GSH by GR was followed by the absorbance of NADPH at 340 nm, and the amount of NADPH consumed was calculated by using its molar extinction coefficient (ε = 6.22 mM^−1^ cm^−1^). The activity of GPx was expressed as the amount of NADPH consumed per minute per milligram of cytosol protein. The amount of cytosol protein was determined by the BCA method.

##### (g) Measurement of total GSH Level

The level of total glutathione was measured as described by Habig et al. [42]. First, a mixture of 100 μL of the cytosol fraction, 50 μL of 4 mM NADPH, 100 μL of 6 unit/mL GR in 500 μL of 10 mM sodium phosphate buffer at pH 7.5 was preincubated at 37 °C for 5 min to convert GSSG to GSH. Then, 50 μL of 10 mM 5,5′-dithiobis-2-nitrobenzoic acid (DTNB) was added to the mixture. The absorbance of 5-mercapto-2-nitrobenzoic acid at 412 nm produced by the reaction of DTNB and GSH was measured.

#### 2.2.3. Effect of ACSO and Sulfides on Liver Function of Normal Rats

Rats were divided into four groups of six rats. ACSO, DADS, and DAS at a dosage of 50 μmol/mL/day were orally administered to normal healthy rats of the corresponding group for seven consecutive days. ACSO was dissolved in DW, while DADS and DAS were dissolved in olive oil for use. Control rats received only DW or olive oil. The rats were subjected to fasting for 24 h following oral administration of ACSO, DADS, and DAS on the seventh day and then sacrificed. The activities of GST and GPx were measured as described in 2.2.2.

#### 2.2.4. ACSO Absorption and Metabolism in the Small Intestine

##### (a) ACSO Absorption from the Small Intestine

Rats were divided into two groups (*n* = 3) designated as control and ACSO. After acclimation, rats were subjected to fasting overnight. The abdomen was opened on a thermal heat table under anesthesia, and the small intestine was ligated to be closed. The blood was collected from the portal vein through a cannula and designated as the sample at 0 min. Then, the ligated small intestine was injected with phosphate buffered salts (PBS) in the control group and with 100 mM ACSO in PBS for the ACSO group. Blood was collected from the portal vein through the cannula 10, 20 and 30 min after this injection. The small intestine was then excised, and the luminal solution was collected and heated at 80 °C for 30 min for measurement of ACSO and pyruvic-acid content. The excised small intestine from the control group was used for analysis of ACSO metabolism.

##### (b) Measurement of ACSO and Pyruvic-Acid Content

For the measurement of ACSO content, the obtained blood or luminal solution was mixed with 1 M hydrogen peroxide and placed on ice for 10 min. The solution was centrifuged at 10,000× *g* at 4 °C for 10 min, and the supernatant was mixed with 0.7 M potassium carbonate and placed on ice for an additional 10 min. Then, the solution was centrifuged at 2300× *g* at 4 °C for 10 min, and the resultant supernatant was kept cool on ice until use. For measurement of ACSO, the supernatant was mixed with 9-fluorenylmethyl chloroformate (Fmoc-Cl), and Fmoc-derivatized compounds were analysed using HPLC (Alliance e2695, Waters, Milford, MA, USA) equipped with an ODS column (Inertsil ODS-4, GL Sciences, Tokyo, Japan) and fluorescent detector (Waters 2475, Waters, Ex. 263 nm, Em. 313 nm).

For the measurement of pyruvic-acid content, the obtained blood or luminal solution was mixed with 1 M hydrogen peroxide and placed on ice for 10 min. The solution was centrifuged at 10,000× *g* at 4 °C for 10 min, and the supernatant was mixed with 6 N HCl. The solution was incubated with 1,2-diamino-4,5-methylenedioxybenzene (DMB), 9 N HCl, 2-mercaptoethanol, and Na_2_S_2_O_4_ at 100 °C for 45 min. After cooling, the supernatant was collected and filtered. The obtained solution containing DMB-derivatized compounds was analyzed with HPLC (Alliance e2695) equipped with an ODS column (Separar C18G, Rikaken, Japan) and fluorescent detector (Waters 2475, Ex.367 nm, Em. 446 nm).

##### (c) Analysis of Volatile Metabolites from ACSO in the Small Intestine

The excised small intestines were rinsed with 0.02 M K_2_HPO_4_-KH_2_PO_4_ buffer at pH 7.5 and homogenized with the same solution before centrifugation at 15,000× *g* at 4 °C for 30 min. The supernatant was centrifuged at 105,000× *g* at 4 °C for 60 min. Ammonium sulfate was added to the obtained supernatant to yield precipitated protein. The concentration of ammonium sulfate was increased stepwise, and the precipitate was recovered at each stage. The protein precipitated by 60–80% ammonium sulfate was collected and dialyzed against 0.02 M K_2_HPO_4_-KH_2_PO_4_ buffer. The dialyzed solution was purified with cation-exchange chromatography (CM Sepharose FF, GE Healthcare, Chicago, IL, USA), and the obtained fraction was mixed with 50 mM ACSO in 0.02 M K_2_HPO_4_-KH_2_PO_4_ buffer at 30 °C for 30 min in a sealed vial. Volatiles in the headspace were collected by solid-phase microextraction using divinylbenzene/carboxen/polydimethylsiloxane fiber (57328-U, Sigma-Aldrich, St. Louis, MO, USA) for 30 min while the solution was continuously stirred. The absorbed volatiles were analyzed by GC-Atomic Emission Detector (HP 6890GC, HP G2350A AED, Agilent technology, Santa Clara, CA, USA) equipped with analytical column (DB-1, J&W Scientific, Folsom, CA, USA). Sulfuric compounds were detected at 181 nm, and carbon compounds were detected at 193 nm.

### 2.3. In vitro Experiments

#### 2.3.1. Cell Culture

Human Caucasian hepatocyte carcinoma HepG2 cells (HB-8065, Lot No: 16K046, passages 10–20, ATCC, Manassas, VA, USA) were grown in Eagle’s Minimum Essential Medium (EMEM) supplemented with 10% (*v*/*v*) fetal bovine serum, 100 U/mL penicillin, and 100 g/mL streptomycin. Cultures were grown at 37 °C under 5% CO_2_. Stock cultures were grown in 75-cm^2^ flasks (Corning, Tokyo, Japan), and media was replaced every 2 days. Cells were routinely subcultured by trypsinization upon reaching 80–90% confluency.

#### 2.3.2. Nrf2 Content in the Nucleus, Cytoplasm, and Whole Cells

A suspension of HepG2 cells at a density of 7 × 10^5^ cells/mL in EMEM medium containing 10% FBS was incubated at 37 °C under 5% CO_2_ for 48 h in a 100-mm dish. The medium was then removed, and EMEM medium containing 1% BSA and 0.25–1.0 mM ACSO was added. The cells were further incubated at 37 °C under 5% CO_2_ for 6 h. After removal of the medium, cells were detached in 5 mL PBS using a cell scraper. The obtained suspension was centrifuged at 300× *g* for 5 min at room temperature to pellet the cells. To obtain whole cells, the precipitate was mixed with lysis buffer and sonicated. The resulting suspension was centrifuged at 1000× *g* at room temperature for 10 min, and then the supernatant was designated as the whole-cell fraction. Nuclei and cytoplasm of HepG2 cells were extracted from the obtained precipitate using the Nuclear Extraction Kit (RayBiotech, Peachtree Corners, GA, USA), according to the manufacturer’s instructions. These samples were used for SDS-PAGE analysis with a 12.5% acrylamide gel. After transfer of protein from the gel, the PVDF membrane was blocked for 30 min in 150 mM NaCl; 10 mM Tris/HCl, pH 7.4; 0.05% (*v*/*v*) Tween 20, and 0.5% (w/v) skim milk powder. PVDF membranes were incubated overnight at 4 °C with primary antibodies raised against Nrf2 (E-AB-32280, Lot No: DK7634, Elabscience, Houston, TX, USA; 1:2000) and β-actin (sc-47778; Lot No: K1418, Santa Cruz Biotechnology, Dallas, TX, USA; 1:1000). After this incubation, membranes were incubated HRP-linked secondary anti-rabbit IgG (7074S, Lot No: 27, Cell Signaling Technology, Danvers, MA, USA; 1:10,000) or anti-mouse IgG (7076S, Lot No: 33, Cell Signaling Technology, 1:5000). Protein was visualized and quantified using ECL Western blot detection system (RPN2235; GE Healthcare, Tokyo, Japan) and Image Lab software (ChemiDoc XRS Plus; Bio-Rad Laboratories, Hercules, CA, USA).

### 2.4. Statistical Analysis

**Figure 3:**
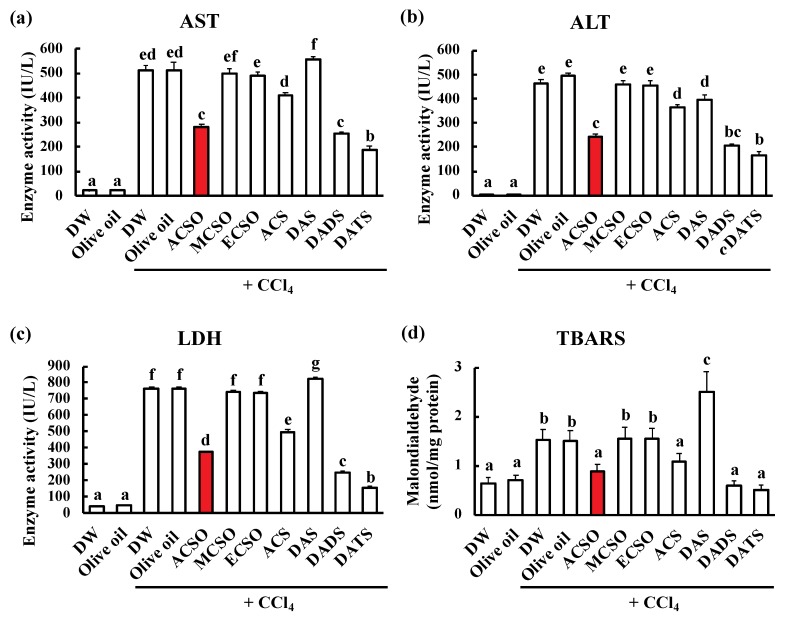
Effect of ACSO, its analogs, and garlic-odor compounds on markers for hepatic injury and oxidative stress. Aspartate transaminase (AST) activity (**a**), alanine transaminase (ALT) activity (**b**), lactate dehydrogenase (LDH) activity (**c**), and the amount of thiobarbituric acid reactive substances (TBARS) (**d**) in the blood of rats with CCl_4_-induced hepatic injury. Each value represents the mean of six rats ± S.E. The different letters in the figures indicate a significant difference between the groups (*p* < 0.01).

**Figure 4:**
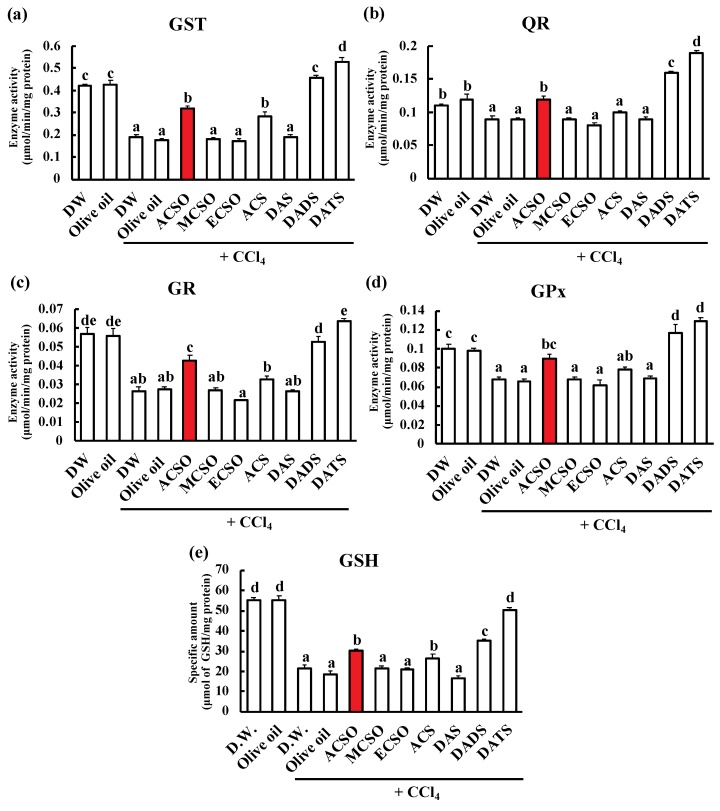
Effect of ACSO, its analogs, and garlic-odor compounds on Phase II and antioxidative enzyme activities and glutathione (GSH) content. Enzymatic activities of (**a**) glutathione-*S*-transferase (GST), (**b**) quinone reductase (QR), (**c**) glutathione reductase (GR), (**d**) glutathione peroxidase (GPx), and (**e**) GSH, in the livers of rats with CCl_4_-induced hepatic injury. Each value represents the mean of six rats ± S.E. The different letters in the figures indicate a significant difference between the groups (*p* < 0.01).

**Figure 5:**
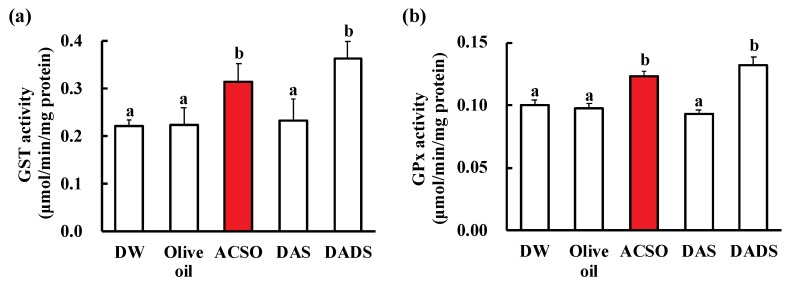
Effect of ACSO and sulfides on liver function. GST activity (**a**) and GPx activity (**b**) in the livers of normal rats after administration of CCl_4_. Each value represents the mean of six rats ± S.E. The different letters in the figures indicate a significant difference between the groups (*p* < 0.01).

**Figure 6:**
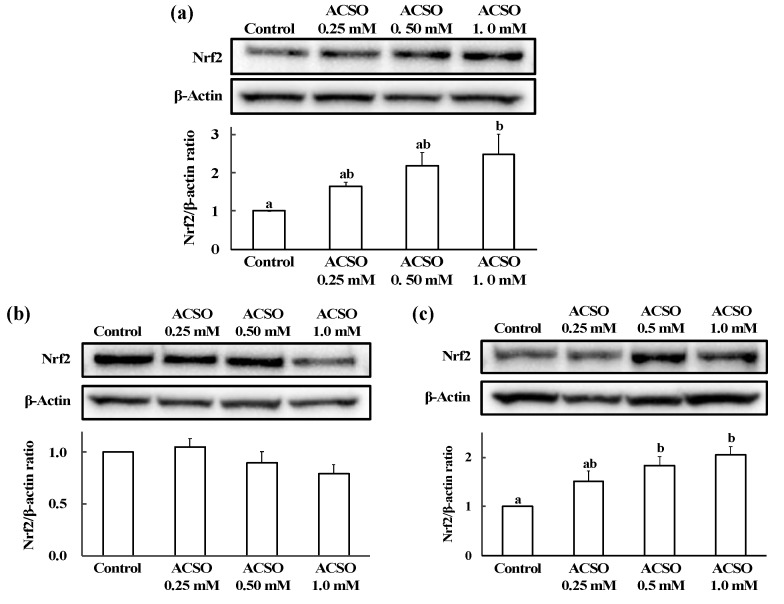
Effect of ACSO on Nrf2 nuclear translocation in HepG2 cells. The expression levels of Nrf2 were analyzed by Western blot analysis in whole cell (**a**), cytoplasm (**b**), and nuclei (**c**) preparations of HepG2 cells. Expression was quantified as the ratio of Nrf2 expression to β-actin expression. Each value represents the mean of three experiments ± S.E. The different letters indicate a significant difference between the groups (*p* < 0.05).

All data were expressed as the mean ± SEM, and the significance of the differences (*p*-values) between groups was evaluated using a one-way ANOVA followed by Duncan’s test (for the animal experiments shown in Figure 3, Figure 4 and Figure 5) or the Tukey-Kramer test (for the cell experiments shown in Figure 6).

## 3. Results

### 3.1. Effect of ACSO, Its Analogs, and Garlic-Odor Compounds on Suppression of Hepatic Injury Induced by CCl_4_

The activities of AST, ALT, and LDH were measured as enzyme markers for acute hepatic injury (Figure 3a–c). AST, ALT, and LDH activities were increased following injection of CCl_4_ in the control groups, and these increases were significantly suppressed by oral administration of ACSO, ACS, DADS, and DATS (*p* < 0.01). In addition, TBARS was measured to evaluate the peroxidation of lipids produced by acute hepatic injury (Figure 3d). TBARS was increased by the injection of CCl_4_ in the control groups, while oral administration of ACSO, ACS, DADS and DATS significantly suppressed the increase in TBARS after the injection of CCl_4_ (*p* < 0.01).

We next measured the effect of oral administration of sulfoxides and sulfides on the enzymatic activities of GST, QR, GR and GPx, and GSH content in the liver after the injection of CCl_4_ (Figure 4). All measured enzyme activities were lower in the control with acute hepatic injury resulting from CCl_4_ than in the control groups without injection of CCl_4_ (*p* < 0.01). GST activities in the ACSO, ACS, DADS, and DATS groups were significantly higher than those in the control, MCSO, ECSO, and DAS groups following injection of CCl_4_ (Figure 4a, *p* < 0.01). QR and GR activities in the ACSO, DADS, and DATS groups were significantly higher than those in the control, MCSO, ECSO, ACS, and DAS groups following injection of CCl_4_ (Figure 4b and c, *p* < 0.01). GPx activities in the ACSO, DADS, and DADS groups were significantly higher than those in the control, MCSO, ECSO, and DAS groups (Figure 4d, *p* < 0.01). GSH content in the ACSO, ACS, DADS, and DATS groups was significantly higher than that in the control, MCSO, ECSO, and DAS groups following injection of CCl_4_ (Figure 4e, *p* < 0.01) and lower than that in the control groups without acute hepatitis. Thus, the activities of the phase II and antioxidative enzymes and the GST content in the ACSO as well as the DADS and DATS groups were higher than those in the control groups even after the induction of hepatic injury.

### 3.2. Effect of ACSO and Sulfides on Liver Function

Figure 5 shows the effect of oral administration of ACSO, DADS, and DATS on GST and GPx activities in the livers of rats without the injection of CCl_4_. GST and GPx activities in the ACSO and DADS groups were significantly higher than those in the control and DAS groups (*p* < 0.01).

### 3.3. Effect of ACSO on Nrf2 Nuclear Translocation in HepG2 Cells

The Nrf2/β-actin ratio in HepG2 cells was determined by Western blot analysis (Figure 6). Nrf2/β-actin ratio in whole HepG2 cells was increased with the addition of ACSO, and the ratio was significantly higher than that in the control group following addition of 1 mM ACSO (Figure 6a, *p* < 0.05). Nrf2/β-actin ratios in the cytoplasm were not significantly different between the groups; however (Figure 6b), the Nrf2/β-actin ratio in the nucleus increased as the concentration of added ACSO increased (Figure 6c). The Nrf2/β-actin ratio in the nuclei of HepG2 cells was significantly higher following the addition of 0.5 and 1 mM ACSO than that in the nuclei of HepG2 cells without the addition of ACSO (*p* < 0.05). 

### 3.4. Absorption and Metabolism of ACSO in the Small Intestine

**Figure 7:**
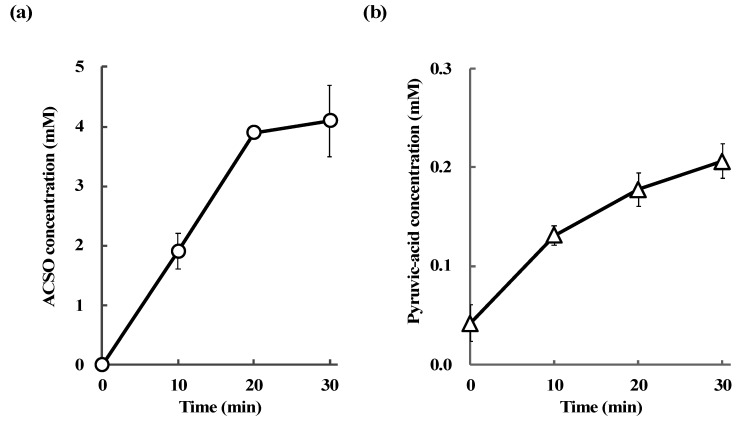
ACSO (**a**) and pyruvic-acid (**b**) concentrations in the portal vein were measured after injection of ACSO into the small intestine. Each value represents the mean of three excited small intestines ± S.E.

The changes in ACSO and pyruvic-acid concentrations in the portal vein after the injection of ACSO into the ligated loop of the small intestine were measured to evaluate the absorption and metabolism of ACSO. Concentrations of both ACSO and pyruvic acid increased as time proceeded (Figure 7). The concentration of ACSO in the portal vein reached 4 mM 30 min after injection of ACSO into the small intestine, while that of pyruvic acid reached 0.2 mM at this time point. ACSO and pyruvic-acid concentrations in the luminal liquid of the small intestine 30 min after injection of PBS into the small intestine were 0 mM and 0.04 mM, respectively. While ACSO and pyruvic-acid concentrations in the luminal liquid of the small intestine 30 min after injection of 100 mM ACSO into the small intestine were 8.02 mM and 0.81 mM, respectively (Table 1). 

**Figure 8:**
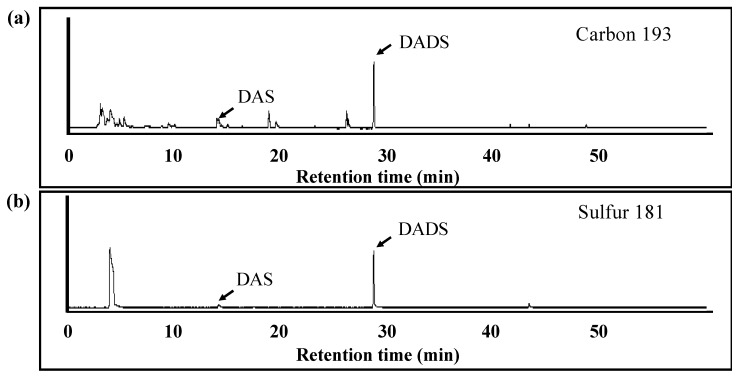
Element chromatograms of volatile compounds obtained from the mixture of ACSO and proteins of the small intestine to assess ACSO metabolism. Carbon was detected at 193 nm (**a**) and sulfur was detected at 181 nm (**b**).

Volatile components produced from the mixture of ACSO and the crude protein extracted from the small intestine were analyzed using solid-phase microextraction and GC-AED in order to further evaluate the metabolism of ACSO in the small intestine. DAS and DADS were detected by GC-AED at retention times of 14 and 29 min, respectively. The relative intensity of DADS was higher than that of DAS (Figure 8).

## 4. Discussion

We prepared ACSO and its analogs MCSO, ECSO, and ACS to compare their effects on the prevention of hepatic injury. MCSO is a minor sulfuric component in garlic and the content is up to 2 mg/g fresh weight [26]. ACS is also a minor sulfuric component: The content is less than 30 μg/g fresh weight, but rich in aged garlic [46]. ECSO is not a naturally occurring compound in garlic. We also examined the garlic odor components DAS, DADS, and DATS in these experiments. ACS [47,48], DADS, and DATS [49,50] have previously been reported to prevent hepatic injury when they are intraperitoneally injected, while DAS did not show such an effect. In this study, we found that oral administration of ACSO suppressed acute hepatic injury induced by CCl_4_ in addition to that of DADS and DATS (Figure 3). The enzymatic activities of hepatic injury markers, such as AST, ALT, and LDH, increased following intraperitoneal injection of CCl_4_; however, these increases were suppressed by oral administration of ACSO as well as that of DADS and DATS. Analysis of the reactive aldehydes produced from lipid hydroperoxides as a malondialdehyde equivalent using the TBARS method revealed that the increase in TBARS after injection of CCl_4_ was remarkably suppressed by oral administration of ACSO, DADS, and DATS to levels approximately the same as that of the control group without CCl_4_ injection. These results suggest that oral administration of ACSO prevented acute hepatic injury induced by CCl_4_ in rats. As MCSO, ECSO, and ACS were not effective, the allyl and sulfoxide groups in ACSO are essential for this preventive effect.

The preventive effect of ACSO on hepatic injury is probably attributable to the induction of detoxifying and antioxidative enzymes, as scavenging of free radicals is crucial for these effects on hepatic injury induced by CCl_4_ and ACSO itself would not function as a free radical scavenger. The activities of GST, QR, GPx, and GR as well as the level of GSH were reduced by the injection of CCl_4_ in the vehicle-treated control group. These reductions in enzymatic activities likely resulted from protein denaturation by radicals, such as CCl_3_, that are produced by CCl_4_. This decrease was suppressed by oral administration of ACSO, DADS, and DATS (Figure 4). The suppressive effect of ACSO stems from its ability to induce GST and GPx activities, as shown in normal rats (Figure 5). The enhanced induction of GST and GPx relieves oxidative stress, preventing protein denaturation and enzyme inactivation and leading to the reduction of oxidized GSH to reduced GSH. Therefore, consecutive oral administration of ACSO induced both phase II and oxidative enzymes, resulting in attenuation of the symptoms of hepatic injury.

Some phase II and antioxidative enzymes, such as GST and GR, are regulated by the Nrf2-Kelch-like ECH-associated protein 1 (Keap1) system. Increases in such enzymes often prevent or attenuate diseases, such as Alzheimer’s disease [51], vascular diseases [52], and cancers [53], that are considered to be caused by oxidative stress. Activation of this pathway is triggered by the release of the Nrf2 transcriptional factor from Keap1, which is a marker of ubiquitination [37]. The release occurs in response to modification of the Cys residues of Keap1 with electrophiles [54]. Translocation of Nrf2 into the nucleus activates transcription of antioxidative and detoxifying enzymes. DADS and DATS have been reported to induce antioxidative and detoxifying enzymes via the activation of the Nrf2-ARE pathway [50]. Since disulfide and trisulfide bonds are readily cleaved by nucleophiles to form covalent linkages with biomolecules [55], DADS and DATS form covalent bonds with Keap1, thus activating the Nrf2-ARE pathway to induce antioxidative and detoxifying enzymes [56]. As the precise mechanism underlying the induction of phase II and antioxidative enzymes by ACSO has not yet been thoroughly investigated, we examined the ability of ACSO to cause translocation of Nrf2 into the nucleus of HepG2 liver cells and demonstrated that the addition of ACSO to these cells induced translocation of Nrf2 into nucleus (Figure 6). Nrf2-ARE pathway controls the expression of a variety of antioxidative enzymes including superoxide dismutase (SOD) and catalase [38], so that ACSO could also increase such enzymes activities not tested in the current study.

Although ACSO was shown to promote Nrf2 nuclear translocation in vitro, the functional molecules to induce phase II and antioxidative enzymes in vivo were not known. One plausible mechanism for the prevention of hepatic injury by oral administration of ACSO was that ACSO itself was absorbed and delivered to the liver and/or metabolized to garlic odor components, which induced phase II and antioxidative enzymes. In vivo experiments showed that the concentrations of both ACSO and pyruvic acid, a metabolite of ACSO, increased in the portal vein after injection of ACSO in the ligated loop of the small intestine (Figure 7). In addition, the pyruvic-acid concentration increased after injection of ACSO in the small intestine (Table 1). These results indicate that ACSO was not only transported to the portal vein but also metabolized to allyl sulfenic acid and pyruvic acid, probably by some enzyme or bacterium present in the small intestine. As allyl sulfenic acid is quite reactive, sulfides such as DADS would be produced in the small intestine. Therefore, ACSO was mixed with crude proteins extracted from the small intestine to examine the production of volatile bioactive sulfides. In these experiments, DAS and DADS were detected as volatile components (Figure 8), and the amount of DADS was greater than that of DAS. These results suggest that orally administered ACSO is partially metabolized to afford DADS, a known inducer of antioxidative and detoxifying enzymes in the liver [49,50]. Taken together, the suppressive effect of oral administration of ACSO on hepatic injury induced by CCl_4_ may stem from the activities of both ACSO itself and its metabolites, including DADS, that induce phase II and antioxidative enzymes in the liver by promoting Nrf2 nuclear translocation. 

In this study, 50 μmol/mL/day of ACSO was orally administered to a rat of approximately 200 g body weight, which simply corresponds to about 45 mg/kg bodyweight/day (molecular weight of ACSO: 177.22). If a person of 50 kg body weight takes the proportional amount, it would become about 2.2 g of ACSO. As fresh garlic contains 14 mg/g weight of ACSO, the amount of garlic corresponding to 2.2 g of ACSO would be approximately 150 g that might be difficult to take daily. However, ACSO is water-soluble and odorless, 2.2 g of ACSO can be easily added to variety of foods. As ACSO is known to enhance richness of taste [27], foods fortified with ACSO may provide both enhanced palatability and health benefit. In the present study, side effects of ACSO were not observed without showing any distinctive changes in the liver weight and the appearance compared to the vehicle group. Although the safety of ACSO to human body should be more precisely investigated, our findings suggest that ACSO has potential to be used as a functional-food additive to prevent diseases caused by oxidative stress.

## 5. Conclusions

Oral administration of ACSO induced phase II and antioxidative enzymes to suppress acute hepatic injury induced by CCl_4_. ACSO was absorbed from the small intestine to the portal vein but was also metabolized to a certain extent in vivo to yield garlic odor components, such as DADS. Because ACSO induced nuclear translocation of Nrf2, ACSO, in addition to DADS, may be an important molecular factor involved in the induction of phase II and antioxidative enzymes and suppression of acute hepatic injury. Oral administration of ACSO may therefore be effective for increasing antioxidative potency and preventing other diseases caused by ROS.

## Figures and Tables

**Table 1:** ACSO and pyruvic-acid concentrations in rat small intestines 30 min after injection of ACSO or PBS into the small intestine.

Sample	ACSO Concentration (mM)	Pyruvic-Acid Concentration (mM)
**PBS**	Not detected	0.04 ± 0.01
**ACSO**	8.02 ± 1.84	0.81 ± 0.07

Each value is the mean of three excited small intestines ± S.E.
